# Analysis of the *HNF4A* isoform-regulated transcriptome identifies *CCL15* as a downstream target in gastric carcinogenesis

**DOI:** 10.20892/j.issn.2095-3941.2020.0131

**Published:** 2021-06-15

**Authors:** Zhen Ni, Wenquan Lu, Qi Li, Chuan Han, Ting Yuan, Nina Sun, Yongquan Shi

**Affiliations:** 1 State Key Laboratory of Cancer Biology & Institute of Digestive Diseases, Xijing Hospital, Air Force Medical University of PLA, Xi’an 710032, China; 2 Department of Gastroenterology, General Hospital of Western Theater Command, Chengdu 610083, China; 3 Department of Gastroenterology, First Affiliated Hospital of Zhengzhou University, Zhengzhou 450052, China; 4 Department of Endocrinology, General Hospital of Western Theater Command, Chengdu 610083, China; 5 Department of Gastroenterology, 989 Hospital of the People’s Liberation Army, Luoyang 471003, China; 6 Department of Gastroenterology, First Affiliated Hospital of Xi’an Medical College, Xi’an 710038, China

**Keywords:** Gastric cancer, carcinogenesis, *HNF4A*, *CCL15*, transcriptomics

## Abstract

**Objective:**

Hepatocyte nuclear factor 4α (*HNF4A*) has been demonstrated to be an oncogene in gastric cancer (GC). However, the roles of different *HNF4A* isoforms derived from the 2 different promoters (P1 and P2) and the underlying mechanisms remain obscure.

**Methods:**

The expression and prognostic values of P1- and P2-*HNF4A* were evaluated in The Cancer Genome Atlas (TCGA) databases and GC tissues. Then, functional assays of P1- and P2-*HNF4A* were conducted both *in vivo* and *in vitro*. High-throughput RNA-seq was employed to profile downstream pathways in P1- and P2-*HNF4A*-overexpressing GC cells. The expression and gene regulation network of the candidate target genes identified by RNA-seq were characterized based on data mining and functional assays.

**Results:**

*HNF4A* amplification was a key characteristic of GC in TCGA databases, especially for the intestinal type and early stage. Moreover, P1-*HNF4A* expression was significantly higher in tumor tissues than in adjacent non-tumor tissues (*P* < 0.05), but no significant differences were found in P2-*HNF4A* expression (P > 0.05). High P1-*HNF4A* expression indicated poor prognoses in GC patients (*P* < 0.01). Furthermore, P1-*HNF4A* overexpression significantly promoted SGC7901 and BGC823 cell proliferation, invasion and migration *in vitro* (*P* < 0.01). Murine xenograft experiments showed that P1-*HNF4A* overexpression promoted tumor growth (*P* < 0.05). Mechanistically, RNA-seq showed that the cytokine-cytokine receptor interactions pathway was mostly enriched in P1-*HNF4A*-overexpressing GC cells. Finally, chemokine (C-C motif) ligand 15 was identified as a direct target of P1-*HNF4A* in GC tissues.

**Conclusions:**

P1-*HNF4A* was the main oncogene during GC progression. The cytokine-cytokine receptor interaction pathway played a pivotal role and may be a promising therapeutic target.

## Introduction

Gastric cancer (GC) remains one of the most common malignancies, and one of the most frequent causes of cancer-related deaths^1^. Due to a lack of specific clinical manifestations in the early stage, most GC patients are generally diagnosed at an advanced stage with an overall 5-year survival of less than 20%, especially in China^2^. Therefore, increasing an understanding of GC pathogenesis may identify novel strategies for more effective treatments.

**Figure 1:**
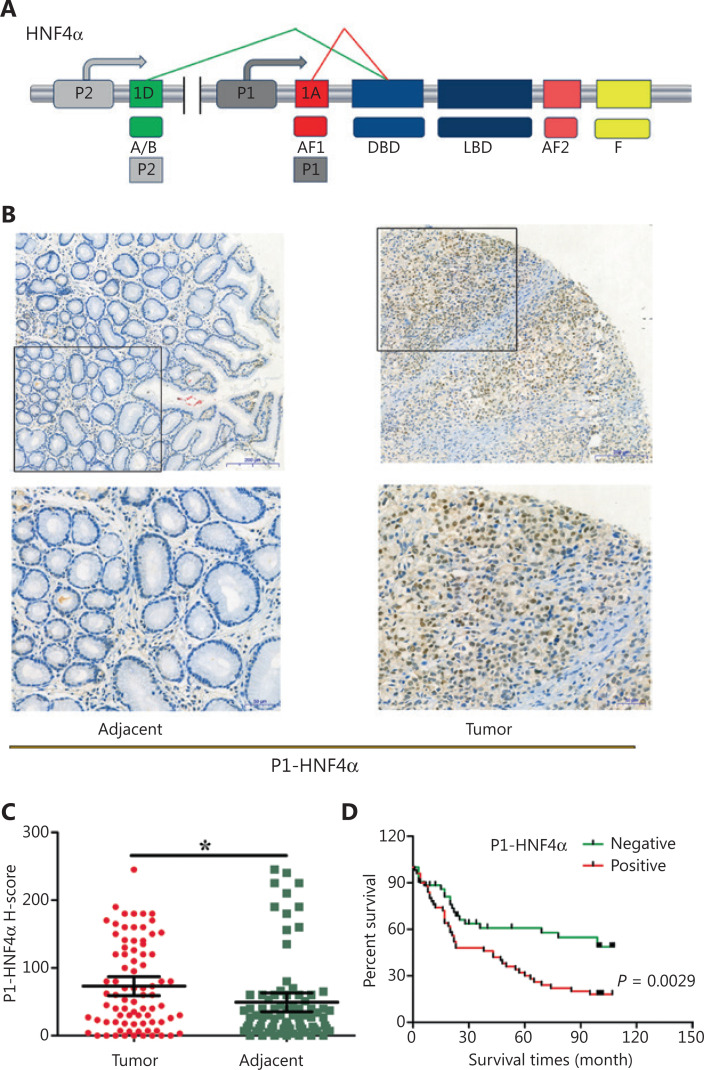
Expression and prognostic value of P1-*HNF4A* in gastric cancer (GC). (A) Schematic of the *HNF4A* gene and its isoforms (P1 and P2). The antigens recognized by isoform-specific (P1 and P2) antibodies are labeled. (B) Representative expression of P1-*HNF4A* in tumor and adjacent non-tumor tissues of GC patients. Scale bars = 200 μm and 50 μm. (C) H-score of P1-*HNF4A* in tumor and adjacent non-tumor tissues using tissue microarray and immunohistochemical analyses (*P < 0.05). (D) The prognostic value of P1-*HNF4A* in GC patients according to negative and positive expression (*P* < 0.01).

Hepatocyte nuclear factor 4α (*HNF4A*) has been previously shown to be an oncogene in GC^3–5^. Mechanistically, *HNF4A* is a target of *NF-*κ*B*^6^, and interacts with the *Wnt* pathway^3^ and controls tumor metabolism required for GC progression through (isocitrate dehydrogenase 1) *IDH1*^5^. However, the specific target genes and downstream signaling pathways of *HNF4A* remain unknown. In addition, a total 9 *HNF4A* isoforms (*HNF4A1-9*), transcribed from P1 or P2 promoters, have been shown to display distinct tissue expressions and functions^7,8^ (**Figure 1A**). Although both P1- and P2-*HNF4A* are present in GC tissues^9^, it remains unclear which splice variant is the most relevant.

In this study, we initially determined the expressions of both P1- and P2-*HNF4A* using TCGA databases and tissue microarrays. Then, we performed transcriptome-wide mapping of both P1- and P2-*HNF4A* targets in GC cell lines. Unbiased analysis revealed that the cytokine-cytokine receptor interaction and *PPAR* signaling pathway genes were most enriched in P1- and P2-dependent manners. We also identified chemokine (c-c motif) ligand 15 (*CCL15*), a member of the chemokine family, as a direct target gene of P1-*HNF4A,* which was required for GC progression.

## Materials and methods

### Cell culture

The human GC cell lines, AGS, MKN45, BGC823, AZ521, N87, and SGC7901, and the normal gastric epithelial cell line, GES-1 (American Type Culture Collection, Manassas, VA, USA) were cultured in RPMI 1640 medium (Thermo Fisher Scientific, Waltham, MA, USA) at 37 °C with a humidified atmosphere of 5% CO_2_ supplemented with 10% fetal bovine serum (Biological Industries, Kibbutz Beit Haemek, Israel) and 1% penicillin-streptomycin solution (Thermo Fisher Scientific). All cell line identities were verified through STR DNA profiling and were negative for mycoplasma contamination.

### Cell transfection

*HNF4A2* (NM_000457) and *HNF4A8* (NM_175914) overexpression lentiviruses and a negative control were constructed by GeneCopoeia (Rockville, MD, USA). Human *CCL15*-shRNA lentivirus and negative controls were constructed by GenePharma Technologies (Shanghai, China) (**Table 1**). Logarithmic phase cells (1 × 10^5^) were seeded in 24-well culture plates. After adherence, lentiviral vectors were added at a final concentration of ˜50 multiplicity of infection (MOI) with 0.5 μg/mL polybrene for 10 h at 37 °C. Fresh medium was replaced 10 h after transfection. The cells were transferred into 25 cm^2^ flasks after reaching 70%–80% confluence. Then, the cell culture medium was replaced every 2–3 days with 2 μg/mL puromycin-containing RPMI 1640 medium.

**Table 1:** The sequences of shRNAs used in this study

Gene symbols	Sequences	5′-3′
Human *CCL15*-121	Sense	CCUUACACAUGAAGAGGCATT
	Antisense	UGCCUCUUCAUGUGUAAGGTT
Human *CCL15*-222	Sense	CAGCCAGAAAGCACUACAATT
	Antisense	UUGUAGUGCUUUCUGGCUGTT
Human *CCL15*-291	Sense	AGCCCTTGAGTCCGGTGTCTT
	Antisense	AAGACACCGGACTCAAGGGCT
Negative control	Sense	UUCUCCGAACGUGUCACGUTT
	Antisense	ACGUGACACGUUCGGAGAATT

shRNAs, short hairpin RNA; *HNF4A*, hepatocyte nuclear factor 4α; *CCL15*, chemokine (C-C motif) ligand 15.

### Cell proliferation assay

Cell proliferation was examined using CCK-8 kits (Thermo Fisher Scientific) according to the manufacturer’s instructions, to determine cell growth. A total of 10^3^ target cells were seeded in 96-well plates for the assays in 100 μL of complete medium. Then, 10 μL of CCK-8 reagent was added to the wells and incubated for 2 h. The cultures were assayed each day for 4 continuous days, and the absorbance was read at 450 nm with a reference wavelength at 650 nm using a Varioskan Flash Multimode Reader (Thermo Fisher Scientific). Each experiment was performed in triplicate and repeated 3 times.

### Cell migration and invasion assays

Cell migration experiments were performed in a transwell chamber covered with polycarbonate membranes (Corning, NY, NY, USA). Cell invasion assays were performed using chambers coated with Matrigel matrix (BD Science, Sparks, MD, USA). Resuspended cells (1 × 10^5^ cells/well) were added to the upper chambers and cultured for 24 h. Migrated or invaded cells were stained with 0.1% Crystal Violet for 20 min at room temperature. Quantification of migrated or invaded cells was performed using ImageJ software (National Institutes of Health, Bethesda, MD, USA).

### Cell cycle assay

Target cells were seeded in 6 cm plates and harvested after reaching approximately 70%–80% confluence. Then, resuspended cells were fixed in 75% ethanol, stained with propidium iodide according to the manufacturer’s protocol (Sigma-Aldrich, St. Louis, MO, USA) and sorted using fluorescence-activated cell sorting (BD Sciences, San Jose, CA, USA). Data were analyzed using ModFit software (BD Sciences).

### Murine xenograft model

Animal experiments were approved by the Institutional Animal Care and Use Committee of the Air Force Medical University of PLA (Approval No. 2018-kq-010). Briefly, 6- to 8-week-old BALB/c nude female mice (Shanghai SLAC Laboratory Animal Co., Shanghai, China) were subcutaneously implanted with 1 × 10^7^ SGC7901 cells transfected with a stable control, P1- or P2-*HNF4A* (*n* = 5). Then, the animals were euthanized and the tumor volumes were calculated using the formula (V = length × width^2^ × 0.5) after 2 weeks. The tumor tissues were then harvested and processed for further hematoxylin and eosin staining and immunohistochemistry (IHC) analysis.

### Protein lysate preparation and Western blot

Cells were digested with RIPA cell lysis buffer (Beyotime Biotechnology, Shanghai, China) with protease and phosphatase inhibitor cocktails (MCE, Shanghai, China) and were collected using a cell scraper. Total protein was extracted and quantified using the bicinchoninic acid method (Thermo Fisher Scientific). Proteins (20–30 μg) were resolved using 10% PAGE (Bio-Rad, Hercules, CA, USA), transferred to a nitrocellulose membrane (Pall Corporation, Port Washington, NY, USA) at 25 V for 30 min, and blocked for 1 h in 10% nonfat milk in 1× TBS/0.1% (v/v) Tween 20 at room temperature. Primary antibodies (*GAPDH*, 1:1,000, #5174; Cell Signaling Technology; *β-actin*, 1:1,000, #4970; Cell Signaling Technology; P1-*HNF4A*, 1:1,000, ab41898; Abcam; P2-*HNF4A*, 1:1,000, PP-H6939-00; R & D Systems; and *CCL15*, 1:1,000, ab219388; Abcam) were added and incubated overnight at 4 °C. Secondary antibodies (goat anti-rabbit IgG, HRP #7074 or goat anti-mouse IgG, HRP, 1:2,000, #7076; Cell Signaling Technology) were incubated at room temperature for 1 h. Signals were detected using Western Lumax Light Sirius HRP substrate reagent (ZETA-Life, San Francisco, CA, USA). All data were normalized to human *β*-actin or *GAPDH*. The bands were scanned using a ChemiDocXRS + Imaging System (Bio-Rad).

### RNA-seq

Total RNA was extracted using the mirVana miRNA Isolation Kit (Ambion, Austin, TX, USA). RNA integrity was evaluated using the Agilent 2100 Bioanalyzer (Agilent Technologies, Santa Clara, CA, USA). The samples with RNA integrity number (RIN) ≥ 7 were subjected to subsequent analysis. The libraries were constructed using the TruSeq Stranded mRNA LT Sample Prep Kit (Illumina, San Diego, CA, USA). Then, these libraries were sequenced using the Illumina HiSeq X Ten platform, and 125 bp/150 bp paired-end reads were generated.

Transcriptome sequencing and analysis were conducted by OE Biotech (Shanghai, China). Raw data were processed using Trimmomatic (Illumina). Differentially-expressed genes (DEGs) were identified using the DESeq (2012) R package (Bioconductor; http://www.bioconductor.org/). A *P* value < 0.05 and fold changes > 2 or fold changes < 0.5 was set as the thresholds for significant differential expression. Hierarchical cluster analysis of DEGs was performed to characterize gene expression patterns. Gene Ontology (GO) enrichment and Kyoto Encyclopedia of Genes and Genomes (KEGG) pathway enrichment analyses of DEGs were performed using R based on the hypergeometric distribution.

### Quantitative real-time PCR

Total RNA was extracted using the RNeasy Mini Kit (QIAGEN, Duesseldorf, Germany), according to the manufacturer’s instructions. In total, 0.5 μg RNA was synthesized into cDNA using the PrimeScript RT Reagent Kit (TaKaRa, Shiga, Japan) and Mir-X mRNA First-Strand Synthesis Kit (TaKaRa) in a 10 μL volume. Real-time PCR was performed on a CFX96 system using TB Green Premix Ex Taq II (TaKaRa) with 2 μL cDNA and 0.8 μL primers in a final volume of 20 μL. The final PCR conditions were as follows: predenaturation at 95 °C for 10 min, followed by 44 cycles at 95 °C denaturation for 10 s, 60 °C annealing for 20 s, and 72 °C extension for 10 s. The target mRNA gene was normalized to human *GAPDH* using the 2^-ΔΔCT^ method. Primer sequences are shown in **Table 2**.

**Table 2:** The sequences of primers used in this study

Gene symbols	Primers	5′-3′
*HNF4A*	Forward	GTTCAAGGACGTGCTGCTCCTA
	Reverse	AGGCATACTCATTGTCATCGATCTG
*CCL15*	Forward	AGGCCCAGTTCACAAATGA
	Reverse	CAGTCAGCAGCAAAGTGAAAG
*GAPDH*	Forward	GCACCGTCAAGGCTGAGAAC
	Reverse	TGGTGAAGACGCCAGTGGA
*CCL15* Promoter 1	Forward	TAACCTGTGAGCCTTCGATG
	Reverse	GTGACCTATGATCACACCACTG
*CCL15* Promoter 2	Forward	GGAAGGTCTTCTAGCCTCTAATC
	Reverse	CACTGCAAACTCCACCTTCTG
*CCL15* Promoter 3	Forward	TAGGCTCAAGTATGTGCACTA
	Reverse	GGTTTGACCTTTGGATTGGG
*CCL15* Promoter 4	Forward	GAGTAGTCTTGGAAAAGGCAAC
	Reverse	GTAATACCAGTACTTTGGGAG

*HNF4A*, hepatocyte nuclear factor 4α; *CCL15*, chemokine (C-C motif) ligand 15.

### Tissue microarrays and immunohistochemistry

GC tissue microarrays, including 98 cases of gastric tumor and adjunct non-tumor tissues, were obtained from Outdo Biotech (HStmA180Su15; Shanghai, China). Patient characteristics are shown in **Table 3**. This study was approved by the Institutional Ethics Committee of Xijing Hospital of the Air Force Medical University of PLA (Approval No. KY20183093-1).

**Table 3:** Clinical characteristics of the patients analyzed using tissue microarrays

Characteristics	*n*	P1-*HNF4A* Negative	Positive	*P*	*CCL15* Low	High	*P*
Age (years)				> 0.05			> 0.05
< 60	32	20	12		16	16	
≥ 60	66	27	39		27	39	
Gender				> 0.05			> 0.05
Male	62	30	32		29	33	
Female	36	16	20		12	24	
TNM stage				> 0.05			> 0.05
I+II	39	17	22		20	19	
III+IV	59	29	30		22	37	
T stage				> 0.05			> 0.05
T1+T2	14	5	9		8	6	
T3+T4	84	42	42		34	50	
LN metastasis				> 0.05			< 0.05
No	26	10	16		16	10	
Yes	72	36	36		25	47	
Distant metastasis				> 0.05			> 0.05
No	89	40	49		38	51	
Yes	9	6	3		3	6	
WHO type				> 0.05			> 0.05
Adenocarcinoma	47	20	27		15	32	
TA	17	5	12		4	13	
MA	10	8	2		5	5	
SRC	14	8	6		6	8	
UC	10	4	6		1	9	

TA, tubular adenocarcinoma; MA, mucus adenocarcinoma; SRC, signet ring cell carcinoma; UC, undifferentiated carcinoma.

Sections were dried overnight at 60 °C. Antigen retrieval was performed by heating in 1× citrate for 2 min and then cooling to room temperature. Endogenous peroxidase activity was blocked using 3% H_2_O_2_ for 10 min. Slides were incubated with mouse anti-human antibodies against Ki67 (1:1,000, ab92742), P1-*HNF4A* (1:100, ab41898; Abcam), P2-*HNF4A* (1:100, PP-H6939-00; R & D Systems), and *CCL15* (1:200, ab219388; Abcam) at 4 °C overnight. The sections were then counterstained with hematoxylin staining and dehydrated.

The slides were scanned and viewed using a Pannoramic Viewer (3DHISTECH, Budapest, Hungary). The staining intensity of P1- and P2-*HNF4A* was semiquantitatively determined using the H-score method^10^. Only unequivocally stained nuclei were considered positive. H-scores < 50 and ≥ 50 were considered as negative and positive, respectively. H-scores < 150 and ≥ 150 were considered low and high expressions, respectively. *CCL15* staining was semiquantitatively performed according to the staining intensity and percentage of positive cells^11^. IHC scores < 6 and ≥ 6 were considered low and high expressions, respectively.

### Dual-luciferase reporter assays

Briefly, 2,000 bp fragments of the human *CCL15* promoter were obtained from Ensembl and predicted in the JASPAR database. The wild-type (WT) and corresponding mutational *CCL15* promoter fragments covering predicted DR1 sites were PCR-amplified and cloned into the firefly luciferase reporter plasmid, pGL3-basic vector (Promega, Madison, WI, USA). Luciferase activity was then detected using a Dual-Luciferase Reporter Assay Kit (Promega) at 48 h after reporter transfection using Lipofectamine 2000 Transfection Reagent (Invitrogen, Carlsbad, CA, USA) in SGC7901 cells. Firefly luciferase activity was normalized to Renilla luciferase activity, and the final data are presented as the fold induction of luciferase activity compared to that of the negative control.

### Chromatin immunoprecipitation (ChIP)

ChIP assays were performed using the EZ ChIP™ Kit (Millipore, Billerica, MA, USA). The cells were cross-linked with 1% formaldehyde for 10 min at 37 °C and quenched with 2.5 M glycine for 5 min at room temperature. DNA was immunoprecipitated from the sonicated cell lysates using *HNF4A* antibody (Abcam, Cambridge, MA, USA) and subjected to PCR to amplify the HNF4 binding site (**Table 2**). The amplified fragments were analyzed on an agarose gel. A nonspecific antibody against IgG served as a negative control.

### Data mining using public databases

The mRNA expression and DNA copy number of *HNF4A* in GC were analyzed within the Oncomine (http://oncomine.org), GEPIA (http://gepia.cancer-pku.cn), and Ualcan (http://ualcan.path.uab.edu) databases. The GEPIA and Ualcan databases were used to analyze candidate gene alterations that were identified as being enriched in RNA-seq. We further used the LinkedOmics (http://www.linkedomics.org), cBioPortal (http://cbioportal.org), and GEPIA databases to characterize the relationships between *HNF4A* and candidate target genes.

### Statistical analysis

All cell culture experiments were performed in triplicate to reduce error and ensure reproducibility. All quantitative data are expressed as the mean ± SEM. Differences between two groups were examined using an unpaired Student’s *t*-test. Differences between multiple groups were compared by one-way analysis of variance with Dunnett’s post hoc tests. All categorical data are expressed as rates. Differences between two groups were examined using the χ^2^ test. Statistical analysis was performed using SPSS 13.0 statistical software for Windows (SPSS, Chicago, IL, USA) or GraphPad Prism 5.0 (GraphPad Software, San Diego, CA, USA). *P* < 0.05 was considered statistically significant.

## Results

### The expression levels of *HNF4A* in TCGA databases

Initially, the mRNA expression and DNA copy number of *HNF4A* in GC were analyzed within the Oncomine, GEPIA, and Ualcan databases. Data in the Oncomine database revealed that mRNA expression and DNA copy number variation of *HNF4A* were significantly higher in GC tissues than in normal tissues in TCGA Gastric, Deng Gastric, Cho Gastric, and DErrico Gastric (*P* < 0.01), especially for intestinal type adenocarcinomas (*P* < 0.01) (**Supplementary Figure S1**). Data in the Ualcan database also showed significantly higher expression levels of *HNF4A* in tumor tissues than in non-tumor tissues (**Supplementary Figure S2A**). Early stages showed significantly higher *HNF4A* expression levels than those in late stages (**Supplementary Figure S2B and S2C**). Finally, data in the GEPIA database also revealed significantly higher *HNF4A* expression levels in tumor tissues than in non-tumor tissues (**Supplementary Figure S3A**). Furthermore, isoform analysis indicated that *HNF4A1* and *HNF4A2* expressions were significantly higher than *HNF4A7* and *HNF4A8* expressions (**Supplementary Figure S3B**). Together, these results showed that *HNF4A* amplification was common in GC, especially in the intestinal type and early stages. In addition, P1-*HNF4A* upregulation was more remarkable than P2-*HNF4A* upregulation.

### The expression levels of P1- and P2-*HNF4A* in gastric tumors and adjacent tissues

Although high *HNF4A* expression has been shown in GC tissues^3–5^, previous studies did not distinguish the functions of different *HNF4A* isoforms. We therefore detected the expression levels of both P1- and P2-*HNF4A* in gastric tumors and adjacent non-tumor tissues using tumor microarrays. The results indicated that P1-*HNF4A* expression was significantly increased in tumor tissues compared with non-tumor tissues (*P* < 0.05) (Figure 1B and 1C). However, no significant difference was noted in P2-*HNF4A* expression (P > 0.05) (**Supplementary Figure S4A and S4B**), although the H-score of P2-*HNF4A* was higher in non-tumor tissues than in tumor tissues (*P* > 0.05) (**Supplementary Figure S4B**). Next, we examined P1-*HNF4A* expression according to cancer clinical features. As shown in **Table 2**, P1-*HNF4A* expression levels showed an upregulation in the I+II, T1+T2, M0, and N0 stages, but no significant difference was noted (*P* > 0.05). Finally, patients with positive P1-*HNF4A* staining showed a significantly shorter survival compared with negative cases (*P* < 0.05) (**Figure 1D**). However, no significant difference was noted between low and high P2-*HNF4A* expression patients (**Supplementary Figure S4C**). Together, these results indicated that P1- and P2-*HNF4A* might play different roles during GC progression. P1-*HNF4A* expression was the main isoform in gastric carcinogenesis and predicted poor prognoses. However, no significant difference was observed in P2-*HNF4A* expression levels.

### P1- and P2-*HNF4A* showed distinct functions both *in vitro* and *in vivo*

**Figure 2:**
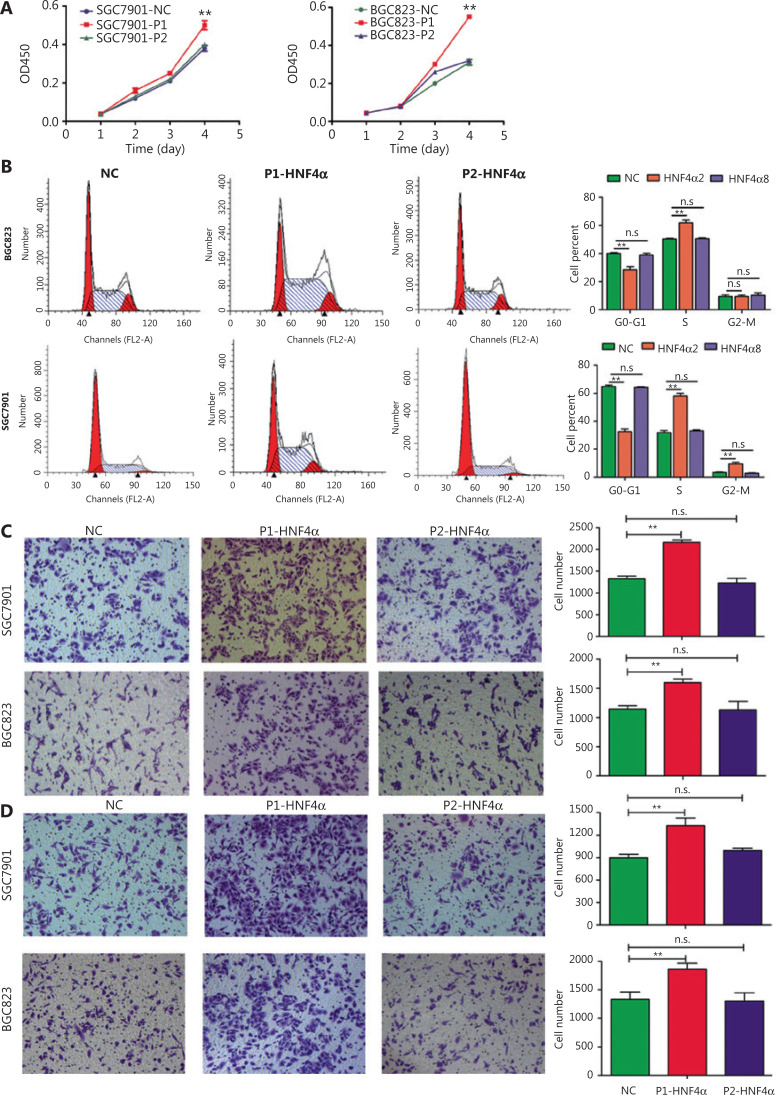
The effects of both P1- and P2-*HNF4A* on malignant phenotypes of gastric cancer cells *in vitro*. (A) P1-*HNF4A* or P2-*HNF4A* overexpression lentivirus were transfected into SGC7901 and BGC823 cells. Cell proliferation was examined using the CCK8 assay. OD_450_ absorbance was detected every day for a continuous 4 days (**P < 0.01, n.s., nonsignificant, *n* = 3). (B) The effects of P1- and P2-*HNF4A* overexpression on SGC7901 and BGC823 cell cycles using a flow cytometry assay (**P < 0.01; n.s., nonsignificant, *n* = 3). (C) The effects of both P1- and P2-*HNF4A* overexpression on SGC7901 and BGC823 cell migrations using transwell experiments. Migrated cells were counted using ImageJ (**P < 0.01; n.s., nonsignificant, *n* = 3) (Crystal Violet staining, 20×). (D) The effects of both P1- and P2-*HNF4A* overexpression on SGC7901 and BGC823 cell invasion using transwell experiments. Invaded cells were counted by ImageJ (**P < 0.01; n.s., nonsignificant, *n* = 3).

We further examined the roles of both P1- and P2-*HNF4A* in GC cells *in vitro* and *in vivo*. First, we detected the expression levels of P1- and P2-*HNF4A* in GC cell lines. Western blot results indicated that P1- and P2-*HNF4A* expression levels showed dramatic differences (**Supplementary Figure S5A**). Then, we directly introduced P1-*HNF4A* (*HNF4A2*) and P2-*HNF4A* (*HNF4A8*) into SGC7901 and BGC823 cells, respectively, 2 cell lines with relatively low P1- and P2-*HNF4A* expressions (**Supplementary Figure S5B**). The CCK-8 assay indicated that P1-*HNF4A* overexpression promoted GC cell proliferation (*P* < 0.01) (Figure 2A). However, no significant difference was noted after P2-*HNF4A* overexpression (P > 0.05) (**Figure 2A**). Furthermore, flow cytometry analysis showed that P1-*HNF4A* overexpression promoted G1/S phase arrest (*P* < 0.01), and P2-*HNF4A* overexpression showed no significant effect on the cell cycle (P > 0.05) (**Figure 2B**). Furthermore, P1- and P2-*HNF4A* promoted *ERK1/2* pathway activation, with a more significant tendency in P1-*HNF4A*-overexpressing cells (**Supplementary Figure S5B**).

**Figure 3:**
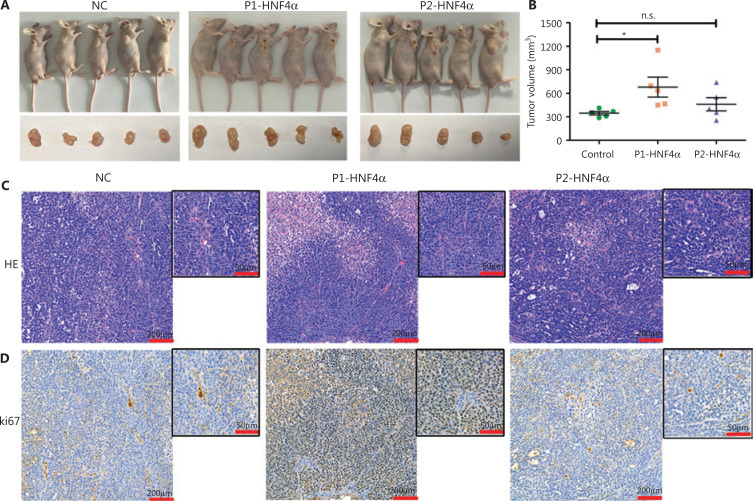
The roles of both P1- and P2-*HNF4A* on gastric cancer (GC) progression *in vivo* using murine xenograft experiments. (A) BALB/c nude mice underwent orthotopic implantation with SGC7901 cells stably transfected by a negative control, P1-*HNF4A,* and P2-*HNF4A* overexpression lentiviruses. (B) The tumors were extracted and measured. Tumor volume was calculated using the formula (V = length × width^2^ × 0.5). The results are expressed as the mean ± SEM (*P < 0.05, n.s., nonsignificant). (C) H&E staining of sections from orthotopic specimens. Scar bars = 200 μm and 50 μm). (D) Ki67 staining of section from orthotopic specimens by immunohistochemistry. Scale bars = 200 μm and 50 μm).

Next, transwell invasion and migration assays also showed that P1-*HNF4A* overexpression facilitated GC cell invasion and migration (*P* < 0.01), but P2-*HNF4A* overexpression showed no effect (P > 0.05) (**Figure 2C and 2D**). Moreover, we performed an *in vivo* xenograft mouse experiment using stably transfected SGC7901 cells. The results showed that the average tumor volume in the P1-*HNF4A* group was significantly larger than that in the control group (*P* < 0.05) (Figure 3A and 3B). However, no significant difference was found in the average tumor volume in the P2-*HNF4A* group compared with the control (P > 0.05) (**Figure 3A and 3B**). In addition, tumors from P1-*HNF4A* overexpression mice showed more significant *Ki67* expression than those from P2-*HNF4A* overexpression mice (**Figure 3C and 3D**).

Taken together, the above *in vitro* and *in vivo* results indicated that P1-*HNF4A* could significantly promote GC progression, and that P2-*HNF4A* showed no significant effect.

### RNA-seq showed distinct pathway and gene enrichment in P1- and P2-*HNF4A*-overexpressing cells

**Figure 4:**
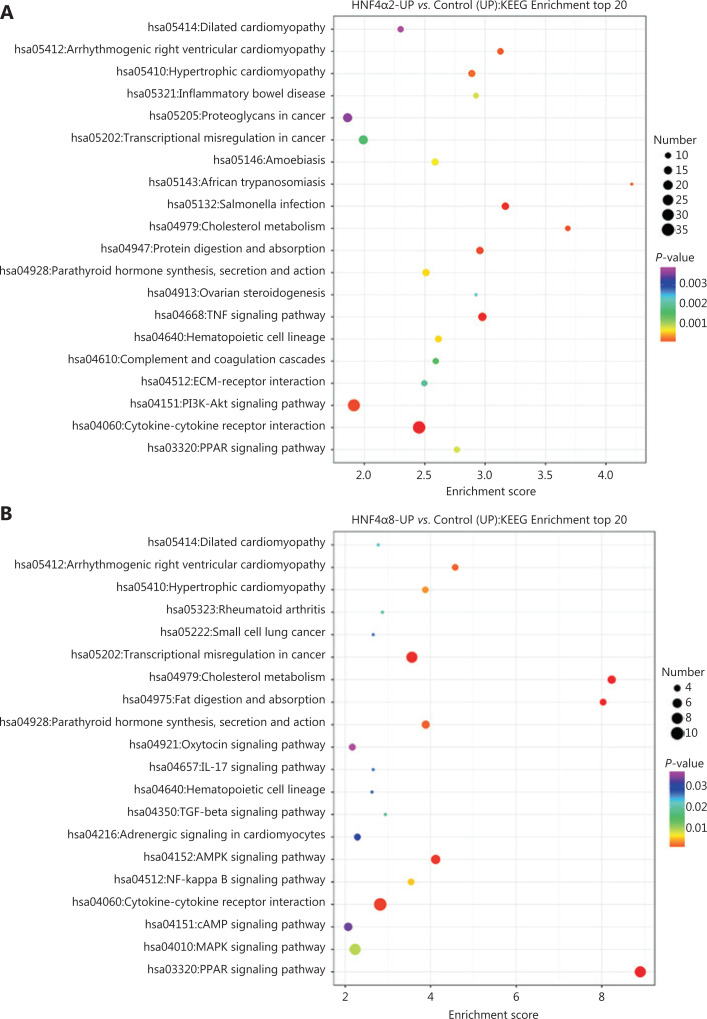
RNA-seq screen of target genes and signaling pathways using downstream P1- and P2-*HNF4A* overexpression in SGC7901 cells. (A) Kyoto Encyclopedia of Genes and Genomes (KEGG) pathway analysis showing enrichment of the top 20 signaling pathways in P1-*HNF4A* overexpression compared to the negative control in SGC7901 cells. (B) KEGG pathway analysis showing enrichment of the top 20 signaling pathways in P2-*HNF4A* overexpression compared to the negative control in SGC7901 cells.

To investigate the underlying mechanisms mediating P1- and P2-*HNF4A* functions, we performed RNA-seq using P1- and P2-*HNF4A*-overexpressing SGC7901 cells. For this purpose, we transfected SGC7901 cells with P1-*HNF4A*, P2-*HNF4A* and negative control lentiviruses. Increased expression of P1- and P2-*HNF4A* upon transfection was confirmed by Western blot (**Supplementary Figure S5B**). Then, we performed RNA-seq to examine the effects of P1- and P2-*HNF4A* on the SGC7901 transcriptome. We found that both P1- and P2-*HNF4A* overexpression resulted in considerable changes in gene expression at the mRNA level (**Supplementary Figure S6A**). Using 2-fold as a cutoff to designate DEGs, we found 310 and 105 genes that were downregulated and 763 and 190 genes that were upregulated in P1- and P2-*HNF4A*-overexpressing cells, respectively (**Supplementary Figure S6B**). There was an overall greater effect in P1-*HNF4A* than in P2-*HNF4A* in terms of the number of dysregulated genes with large fold changes, which could be because P1-*HNF4A* typically has a more potent transactivation function than P2-*HNF4A*. KEGG pathway analysis of the differentially regulated genes showed that there was a significant upregulation of genes involved in the cytokine-cytokine receptor interaction, and a decrease in genes involved in the calcium signaling pathway in P1-*HNF4A* cells (**Figure 4A**). In P2-*HNF4A* cells, there was a significant upregulation of genes involved in the *PPAR* signaling pathway and a decrease in genes involved in valine, leucine, and isoleucine biosynthesis (**Figure 4B**).

### *CCL15* is a direct target of P1-*HNF4A* and is functionally required in GC

To test the deregulated genes involved in the cytokine-cytokine receptor interaction pathway in GC development, we examined the expression of these genes within TCGA databases. Data in GEPIA revealed that the expressions of *CCL5*, *CCL15*, *CCL20*, *CSF2RA*, *CXCL1*, *CXCL8*, *CXCL16*, *CXCR4*, *GDF15*, *IL1RN*, *IL2RB*, *IL2RG*, *IL22RA1*, *INHBA*, *TNFRSF1B*, and *TNFRSF14* were significantly higher in GC tissues than in normal tissues (**Supplementary Figure S7**). Data from Ualcan revealed that the expressions of *BMP2*, *CCL5*, *CCL15*, *CCL20*, *IL8*, *CD70*, *CSF2RA*, *CXCL1*, *CXCL16*, *CXCR4*, *TGFB2*, *GDF15*, *IL11*, *IL1R1*, *IL1RN*, *IL13RA2*, *IL32*, *IL2RB*, *IL2RG*, *IL22RA1*, *IL18R1*, *IL18RAP*, *INHBA*, *INHBB*, *INHBE*, *TNFRSF9*, *TNFRSF1B*, and *TNFRSF14* were significantly higher in GC tissues than in normal tissues (**Supplementary Figure S8**).

**Figure 5:**
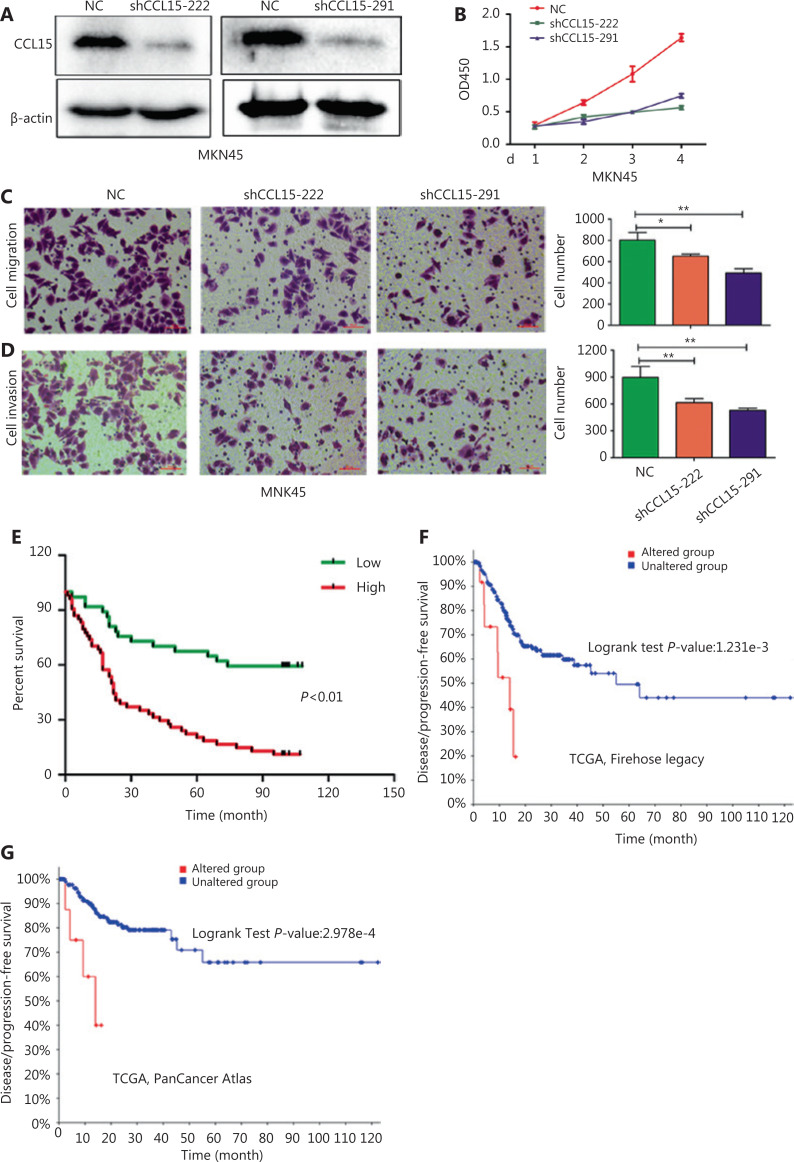
The effects of *CCL15* on malignant phenotypes of gastric cancer (GC) cells *in vitro*. (A) Negative control and sh-*CCL15* lentivirus were transfected in MKN45 cells and *CCL15* protein expression was detected by Western blot. *β-actin* was used as internal control. (B) MKN45 cell viability infected with lentivirus expressing shRNAs targeting *CCL15* or control shRNA was examined by CCK8, and OD_450_ was detected at the indicated time (**P < 0.01, *n* = 3). (C, D) Cell migration and invasion was performed using transwell assays. Migrated (C) or invaded (D) cells were counted by ImageJ (*P < 0.05, **P < 0.01, *n* = 3) (Crystal Violet staining, 20×). (E) The prognostic value of *CCL15* in GC patients using tissue microarrays. (F) The prognostic value of *CCL15* in GC patients of the Firehose legacy cohort using the cBioPortal database (Z-Score = 2). (G) The prognostic value of *CCL15* in GC patients of the PancancerAtlas cohort using the cBioPortal database (Z-Score = 2).

Among the aforementioned gene deregulations in GC, we focused on chemokines, including *CCL15*, the role of which remained unclear in GC progression. Then, we examined the *in vitro* role of *CCL15* on GC cell proliferation and invasion. Initially, we detected the mRNA expression of *CCL15* in the normal gastric epithelial cell line, GES-1, and the GC cell lines, BGC823, MKN45, AZ521, SGC7901, and AGS. Notably, the mRNA level of *CCL15* in GC cells far surpassed that in GES-1 cells (**Supplementary Figure S9**). Then, shRNA-mediated *CCL15* knockdown cells were generated in MKN45, a cell line with relatively high *CCL15* expression (**Figure 5A**). Significant reductions in the proliferation rate were observed in sh*CCL15* cells compared with control cells using the CCK-8 assay (*P* < 0.01) (**Figure 5B**). Significantly reduced cell migration and invasion were also observed in sh*CCL15* cells compared with control cells in the MKN45 cell line (*P* < 0.01) (**Figure 5C and 5D**). To determine whether *CCL15* was also upregulated in primary GCs, we analyzed *CCL15* expression using a GC tissue microarray. The results indicated that *CCL15* expression was significantly higher in tumors than in paracancerous tissues (*P* < 0.01) (**Supplementary Figure S10A and S10B**). Subgroup analysis indicated that patients with high *CCL15* expression showed more lymph node metastasis than those with low *CCL15* expression (*P* < 0.05) (**Table 3**). Survival analysis showed that patients with high *CCL15* expression had a poorer prognosis than patients with low *CCL15* expression (*P* < 0.01) (**Figure 5E**). We further explored the prognostic value of *CCL15* in the cBioportal database. High *CCL15* expression showed poorer prognosis in both the Firehose legacy and PanCancer Atlas groups (**Figure 5F and 5G**). In summary, these results supported *CCL15* as an oncogene in GC progression.

**Figure 6:**
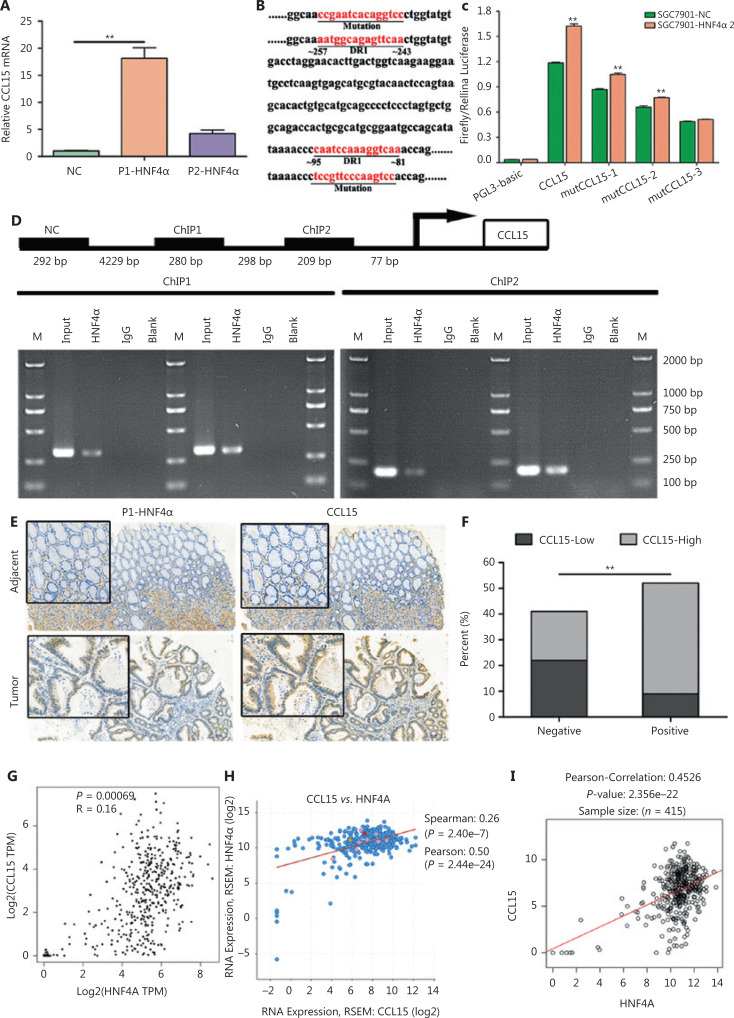
*CCL15* is a direct target of P1-*HNF4A* in gastric cancer. (A) Relative CCL15 mRNA was examined by qRT-PCR in MKN45 cells transfected with the control, P1-*HNF4A*, and P2-*HNF4A* overexpression lentiviruses. *GAPDH* was used as internal control (**P < 0.01, *n* = 3). (B) *CCL15* promoter fragment (˜2,000 bp) from Ensemble was predicted using JASPAR. Reporter genes containing the classical DR1 motifs or mutational sequences were constructed. (C) Negative control (NC) and the *HNF4A2* overexpression plasmid were transiently transfected with *CCL15* promoter reporter constructs with the DR1 motif or mutation for 24 h, and luciferase activity was assayed thereafter. The promoter activity was expressed as fold-induction (means ± SEM) compared to that of the NC, *n* = 3. (**P < 0.01). (D) A chromatin immunoprecipitation assay was performed to show the direct binding of *HNF4A2* to the *CCL15* promoter. M, Marker. (E) Paraffin sections of human gastric cancer tissues and adjacent non-tumor tissues were immunostained with anti-P1-*HNF4A* and anti-*CCL15* antibodies. H&E staining. Scale bars = 200 μm and 50 μm. (F) The percent of *CCL15*-low and *CCL15*-high expression cases in P1-*HNF4A* negative and positive tissues, respectively according staining score (**P < 0.01). (G) The correlation of *CCL15* and *HNF4A* in the GEPIA database. (H) The correlation of *CCL15* and *HNF4A* in the cBioPortal database. (I) The correlation of *CCL15* and *HNF4A* in the LinkedOmics database.

To check if *CCL15* acted as a downstream target, we performed qRT-PCR. The results indicated that both P1- and P2-*HNF4A* could significantly upregulate *CCL15* expression, with P1-*HNF4A* being more prominent (*P* < 0.01) (**Figure 6A**). Then, we examined the promoters of *CCL15* using JASPAR. The results showed that the promoter of *CCL15* contained two predicted DR1 motifs (AGGTCAnAGGTCA) (**Figure 6B**). Next, we constructed luciferase reporter gene fragments covering the two DR1 sequences (wild-type and mutant) (**Figure 6B**). The results revealed that *HNF4A2* positively regulated the *CCL15* promoter containing the two DR1 sites between ˜81˜95 and ˜243˜257 (**Figure 6B and 6C**). Chromatin immunoprecipitation (ChIP) assays further confirmed that *HNF4A2* bound to the speculative sites (˜81˜95 and ˜243˜257) on the *CCL15* promoter in SGC7901 cells (**Figure 6D**). Furthermore, to determine the clinical relevance, we divided the GCs into P1-*HNF4A*-negative and P1-*HNF4A*-positive subgroups according to the H-score. *CCL15* high expression cases in the P1-*HNF4A*-positive group were found to be significantly higher than those in the P1-*HNF4A*-negative group (*P* < 0.01) (**Figure 6E and 6F**). We further determined the correlation between *HNF4A* and *CCL15* in TCGA databases. Both data in the GEPIA, cBioportal, and LinkedOmic databases revealed that HNF4A was positively correlated with *CCL15* (**Figure 6G, 6H, and 6I**).

## Discussion

Accumulating evidence has revealed the vital role of *HNF4A* in GC initiation and progression. However, the functions of different *HNF4A* isoforms and underlying molecular mechanisms in GC cell growth and invasion have not been completely clarified. In this study, we demonstrated that P1-*HNF4A* was the main isoform upregulated in GC progression. Furthermore, we revealed that the cytokine-receptor pathway was one of the key downstream signaling pathways. We also demonstrated that *CCL15* was the most abundant chemokine downstream of P1-*HNF4A* in human GC, with significant prognostic value. These findings revealed complex tumor-promoting effects shaped by the *HNF4A*-*CCL15* axis in human GC.

*HNF4A* is a highly conserved member of the nuclear receptor superfamily, and regulates genes involved in hepatocyte differentiation^12,13^. It also plays key roles in kidney^14^, pancreas^15^, and gut^16^ development, and regulates the expression of genes involved in metabolism^17,18^, homeostasis^19–21^, differentiation^22^, and immune response^23,24^. The roles and functions of *HNF4A* in HCC and CRC have been clearly defined^25–27^. In contrast, the role of *HNF4A* in stomach development and GC has only preliminarily been investigated. Recent studies showed that *HNF4A* is functionally required for GC survival by interacting with the *Wnt* and *NF-*κ*B* pathways and regulating tricarboxylic acid (TCA) cycle metabolism^3,5,6^. In contrast, another recent study showed that the tumor suppressor *ITLN1* promoted *HNF4A* expression by repressing *NF-*κ*B* in GC^28^, and high HNF4A expression showed improved survival outcomes^28^. Similar inconsistent results have also been observed in CRC^27,29^. These paradoxical conclusions indicated that the functions of *HNF4A* were characterized with time and spatial distribution characteristics. The expression of *HNF4A* might be dynamically regulated during GC initiation and progression. Our preliminary results found that *HNF4A* was upregulated in GC, especially in the early stage, which supported the oncogenic roles of *HNF4A*.

A total of nine *HNF4A* splice variants have been generated via two alternative promoters (P1 and P2 promoters) with tissue- and cell-specific expression patterns^30,31^. P1-driven *HNF4A* includes *HNF4A1-6* isoforms and P2-driven *HNF4A* includes *HNF4A7-9* isoforms respectively^30^. P1-driven *HNF4A* is expressed in the adult liver^32^ and kidney^7^, whereas P2-driven *HNF4A* is expressed in the fetal liver, the adult stomach^7^ and pancreas^30^. Both P1- and P2-*HNF4A* are expressed in the adult colon and intestine, but with distinct location distributions^30^. The unique expression patterns of P1- and P2-driven *HNF4A* suggested distinct functional roles of different *HNF4A* isoforms under physiological conditions. Furthermore, previous research has demonstrated that *HNF4A* isoforms showed significant metabolic differences and varied susceptibilities to colitis-associated colon cancer in exon-swap mice^33,34^. In gastric carcinogenesis, both P1- and P2-*HNF4A* were positive in gastric precancerous lesions and tumor tissues^9,35^, which suggests that the *HNF4A* gene may exhibit oncogenic activity in GC. However, the relative contributions of the different *HNF4A* isoforms (P1- and P2-*HNF4A*) have not been determined.

P1-*HNF4A* acts as a tumor suppressor both in HCC and CRC, inhibiting cell proliferation and the inflammation pathway^25,34^. In contrast, P2-*HNF4A* promotes colitis and colitis-associated colon cancer development^34^. Previous studies showed that stomach tissues were positive for only P2-*HNF4A*^7^. In contrast, Moore et al.^19^ indicated that *HNF4A*, especially P1-*HNF4A*, regulated gastric epithelium homeostasis and was necessary for the maintenance of ZC secretory architecture. In this study, we demonstrated that P1- and P2-*HNF4A* exhibited significantly different functions during GC progression. However, unlike in HCC^36^ and CRC^37^, P1-*HNF4A* was the main oncogene, while P2-*HNF4A* expression was irrelevant to GC. Previous studies have demonstrated that P1-*HNF4A* plays key roles in both intestinal epithelium and liver cell development and differentiation^16,23^, so it is possible that tissue specific transcription factors might suppress tumorigenesis. In contrast, ectopic expression of lineage-survival oncogenes may promote cancer by reactivating early developmental programs. These results were consistent with the high expression of P1-*HNF4A* in intestinal-type GC^4^, so we proposed that the function of *HNF4A* was tissue-specific, which might account for the distinct roles of P1- and P2-*HNF4A* in different cancer development. Furthermore, integration of RNA-seq data suggests that this functional difference is due to differential expression of certain target genes, with *HNF4A2* upregulating genes involved in cell proliferation and inflammation, and *HNF4A8* upregulating genes involved in metabolism. In addition, P1-*HNF4A* typically has a more potent transactivation function than P2-*HNF4A*, which displays a similar pattern as in CRC.

Recent evidence suggests the involvement of *HNF4A* in the pathophysiology of inflammatory diseases, such as inflammatory bowel diseases^24,38,39^. *HNF4A* has also been shown to interact with the *IL-6/STAT3* and *NF-*κ*B* pathways^6,25^. Additionally, *HNF4A* was involved in *IL-1β* signal transduction through the regulation of *IL-1R*^6^. These results indicated that inflammation regulation might be a potential molecular mechanism involving the functional roles of *HNF4A*. In this study, we identified *CCL15* as a direct P1-*HNF4A* target gene that promoted GC cell proliferation and invasion. *CCL15* has been shown to play a critical role in tumor progression in various cancer types, including liver and colorectal cancers^40–42^. However, the role of *CCL15* in GC remains unclear. Accumulating evidence has suggested that *CCL15* may have a crucial role in the progression of tumor cells via the CC chemokine receptor^42,43^. Previous studies have preliminarily indicated that *CCL15* expression is higher in gastric tumor tissues than in normal tissues^44,45^. In human CRC cells, loss of *SMAD4* leads to the upregulation of *CCL15* expression^40,42,43^. Additionally, in human liver cancer cells, both inflammatory factors and epigenetics regulate *CCL15* expression^41^. Mechanistically, *CCL15* could promote tumor progression through the only receptor, *CCR1*, which is expressed by both immune cells such as monocytes^41^, lymphocytes^46^, neutrophils^40^, eosinophils^47^ and tumor cells^41,48^. Our results revealed that *CCL15* expression was higher in GC tissues than in normal tissues, and predicted a poor prognosis. *CCL15* knockdown significantly repressed GC cell proliferation, which indicated that *CCL15* might promote GC cell proliferation in an autocrine manner (**Supplementary Figure S11**). Whether *CCL15* promotes GC progression through recruitment of *CCR1* immune cells in a paracrine manner requires further exploration. More importantly, our results indicated the positive correlation of P1-*HNF4A* with *CCL15* in a subset of GC patients. Thus, aberrant P1-*HNF4A* expression in certain GC patients might provide a possible clue for the development of an effective therapy by *CCL15* signaling pathway blockade.

## Conclusions

In summary, our study preliminarily showed the biological and clinical significance of different *HNF4A* isoforms in GC. Compared to P2-*HNF4A*, P1-*HNF4A* might be the main driver oncogene in GC progression. Mechanistically, P1-*HNF4A* directly regulated the cytokine-receptor pathway, thereby promoting tumor growth and progression. *CCL15* could be regarded as a biomarker to guide therapy in GC patients with a high expression of P1-*HNF4A*, and pharmaceutical intervention of the *CCL15* signaling pathway may provide a promising strategy to improve the prognoses of GC patients.

## Data Availability

The RNA-seq datasets generated and analyzed during the current study are available in the NCBI SRA repository (PRJNA612930).
