# Supplementary material for: Analysis of the HNF4A isoform-regulated transcriptome identifies CCL15 as a downstream target in gastric carcinogenesis
Source: Cancer Biol Med. 2021 Jun 15;18(2):530–46. doi: 10.20892/j.issn.2095-3941.2020.0131 (PMC8185874; doi:10.20892/j.issn.2095-3941.2020.0131)
Supplement: Supplementary file 1 [file cbm-18-530-s001.pdf]

Supplementary materials

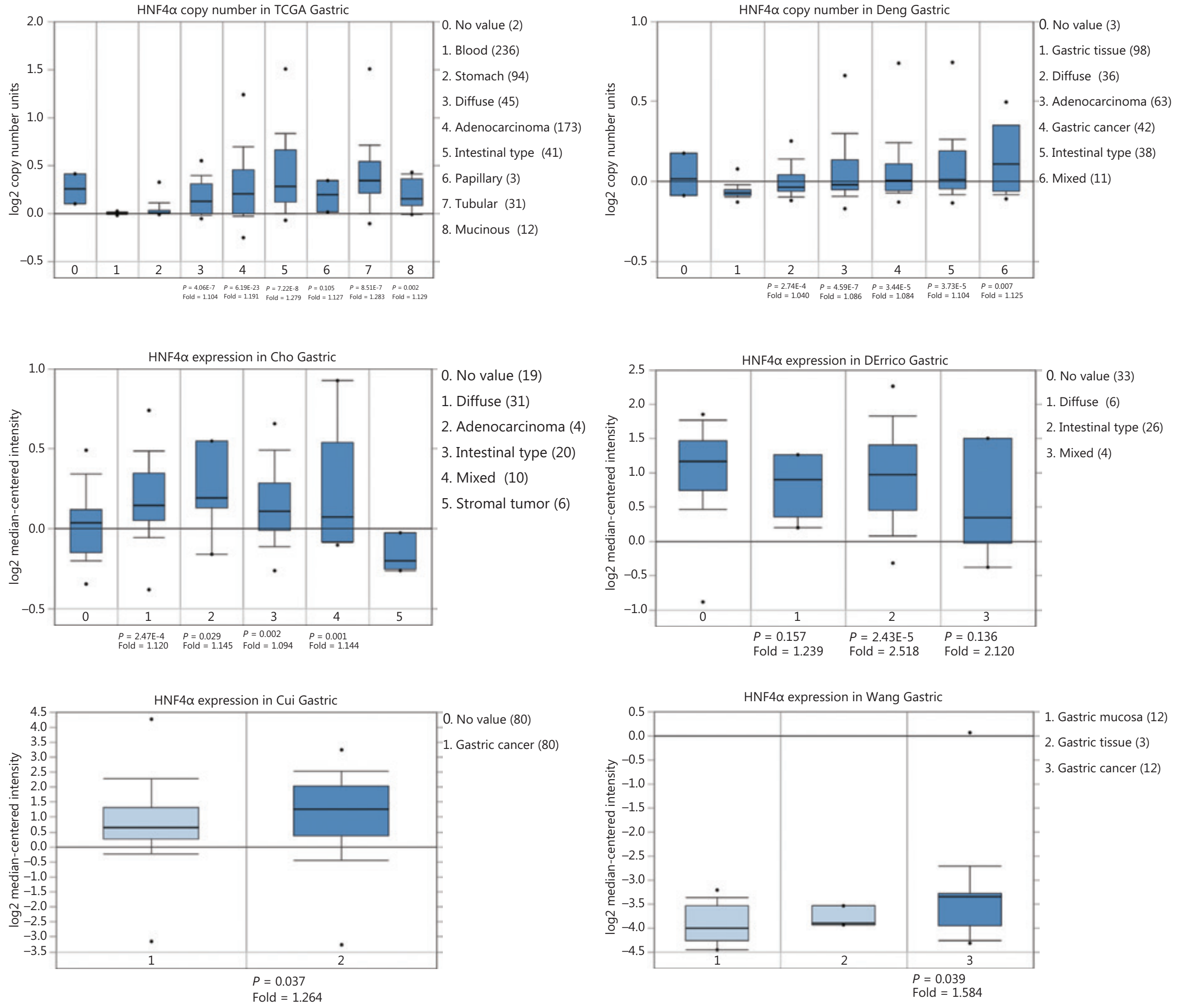

Figure S1 The expression of *HNF4A* in the Oncomine database including TCGA Gastric, Deng Gastric, Cho Gastric, DErrico Gastric, Cui Gastric, and Wang Gastric cohorts.

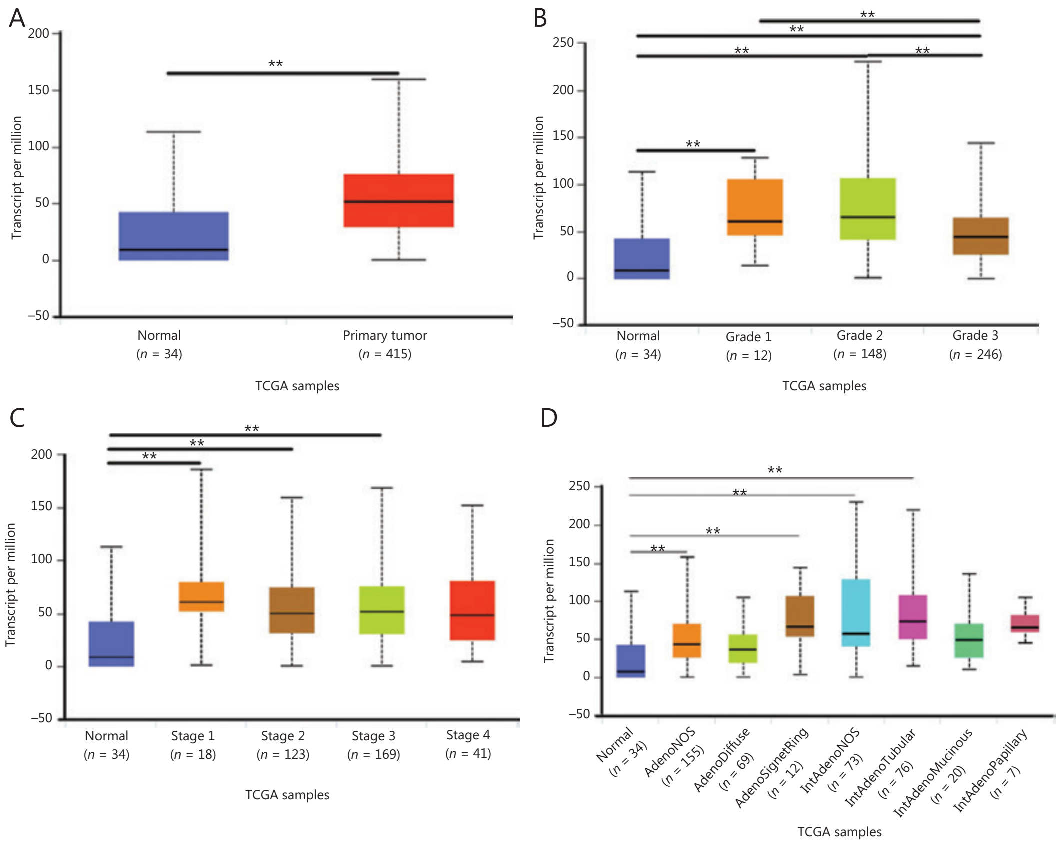

Figure S2 The expression of *HNF4A* in the Ualcan database according to tumor (A), grade (B), stage (C), and tumor type (D) (**P < 0.01).

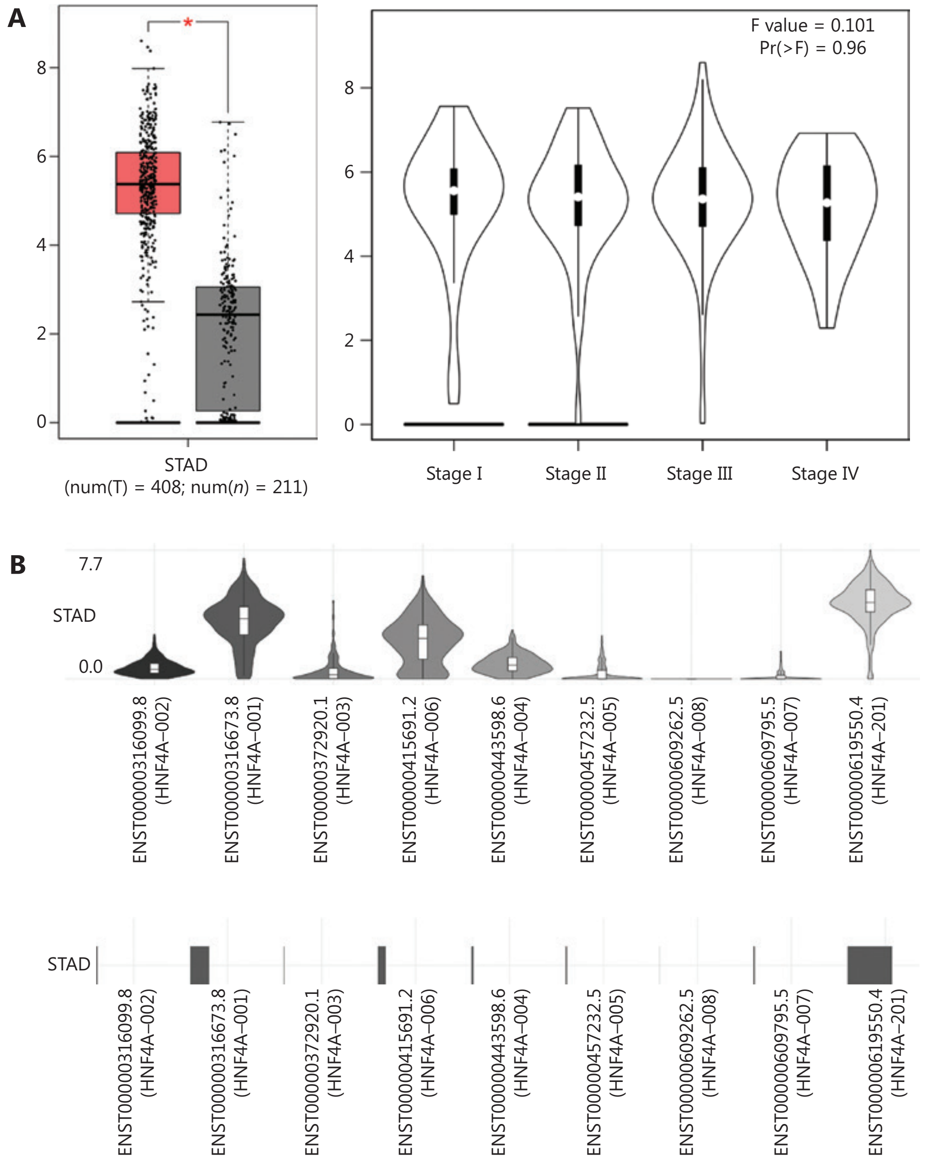

Figure S3 The expression of *HNF4A* in the GEPIA database. (A) The expression of *HNF4A* in tumor and non-tumor cells (*P < 0.05). (B) The expression of different *HNF4A* isoforms in stomach adenocarcinomas.

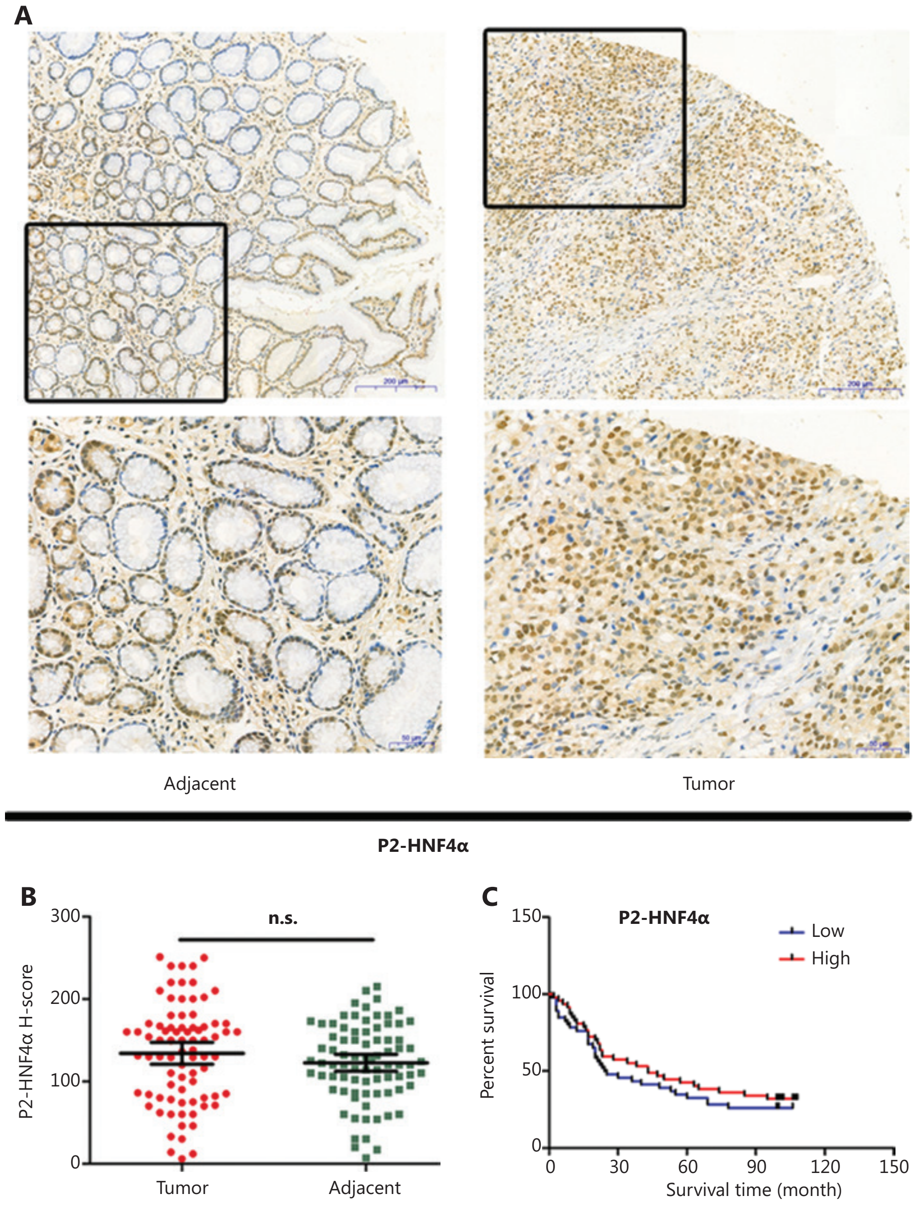

Figure S4 Expression and prognostic value of P2-*HNF4A* in gastric cancer (GC). (A) Representative pictures of the expression of P2-*HNF4A* in tumor and adjacent non-tumor tissues of GC patients. Scale bars = 200 μm and 50 μm. (B) H-score of P2-*HNF4A* in tumor and adjacent non-tumor tissues using tissue microarray using immunohistochemical analysis (n.s., nonsignificant). (C) The prognostic value of P2-*HNF4A* in GC patients according to low and high expressions (P > 0.05).

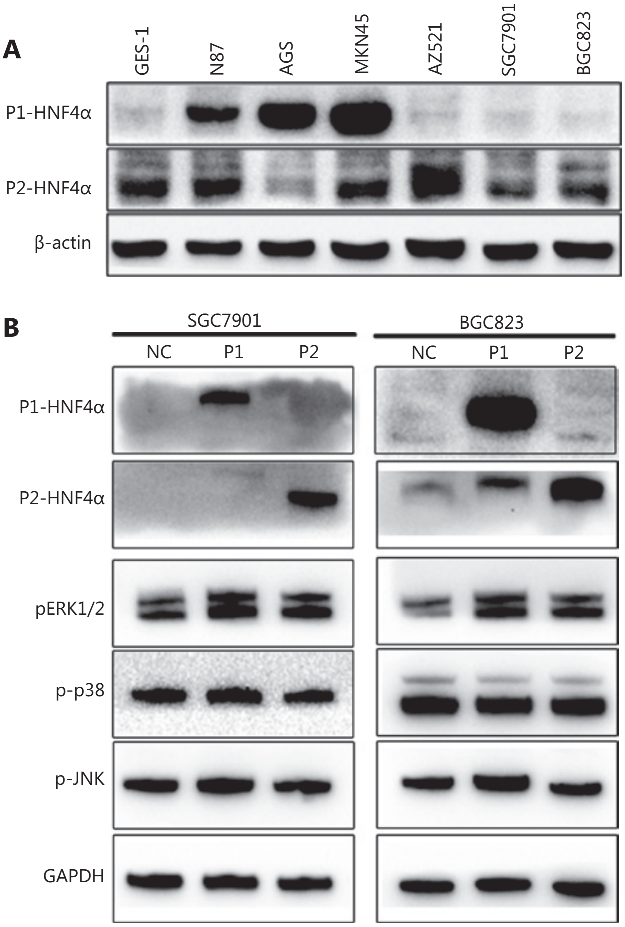

Figure S5 The effects of P1- and P2-*HNF4A* on mitogen-activated protein kinase (*MAPK*) signaling activation. (A) The relative protein expression levels of P1- and P2-*HNF4A* in normal gastric epithelial cell line (GES-1) and gastric cancer cell lines (SGC7901, N87, AGS, AZ521, MKN45 and BGC823) were detected by Western blot. β-actin was used as internal control. (B) The protein expression of *MAPK* in SGC7901 and BGC823 after P1- and P2-*HNF4A* overexpression was detected by Western blot. *GAPDH* was used as an internal control.

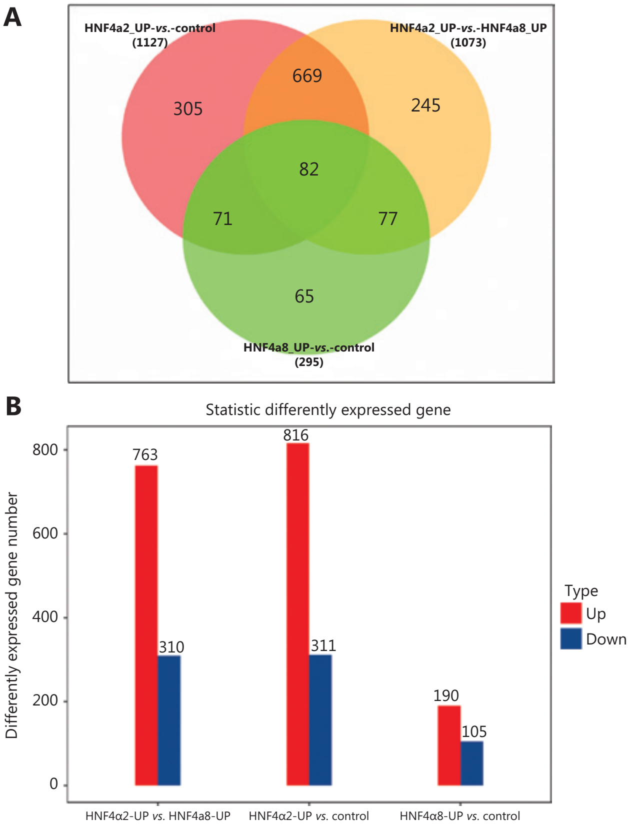

Figure S6 RNA-seq screen of target genes downstream of P1- and P2-*HNF4A* overexpression in SGC7901 cells. (A) A Venn diagram showing the overlap between genes dysregulated by overexpression of P1-*HNF4A* or P2-*HNF4A*. (B) Statistics of differently-expressed gene numbers between negative control, P1-*HNF4A* or P2-*HNF4A* overexpression SGC7901 cells.

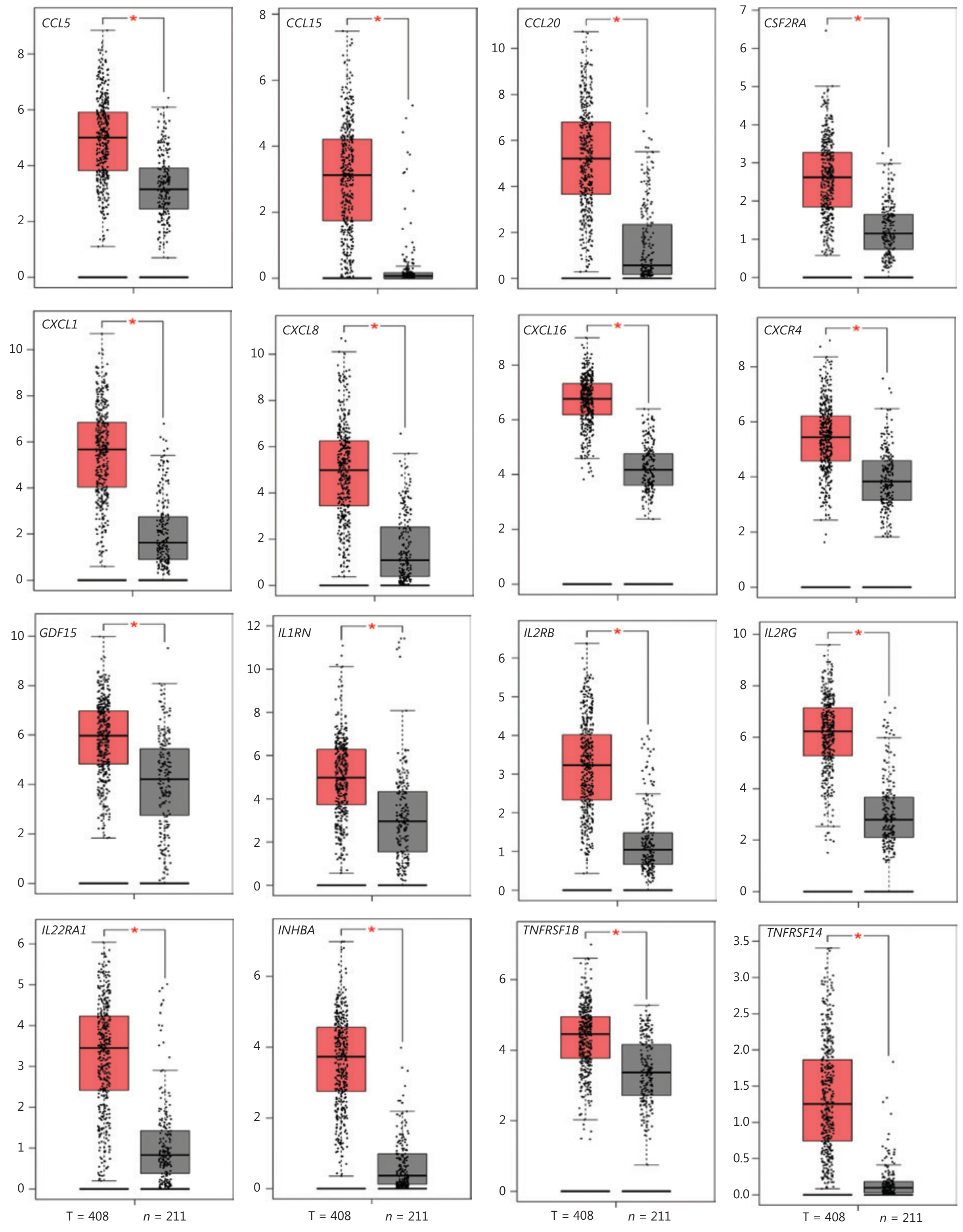

Figure S7 The expressions of *CCL5*, *CCL15*, *CCL20*, *CSF2RA*, *CXCL1*, *CXCL8*, *CXCL16*, *CXCR4*, *GDF15*, *IL1RN*, *IL2RB*, *IL2RG*, *IL22RA1*, *INHBA*, *TNFRSF1B*, and *TNFRSF14* in stomach adenocarcinomas (STAD) in the GEPIA database (**P* < 0.05).

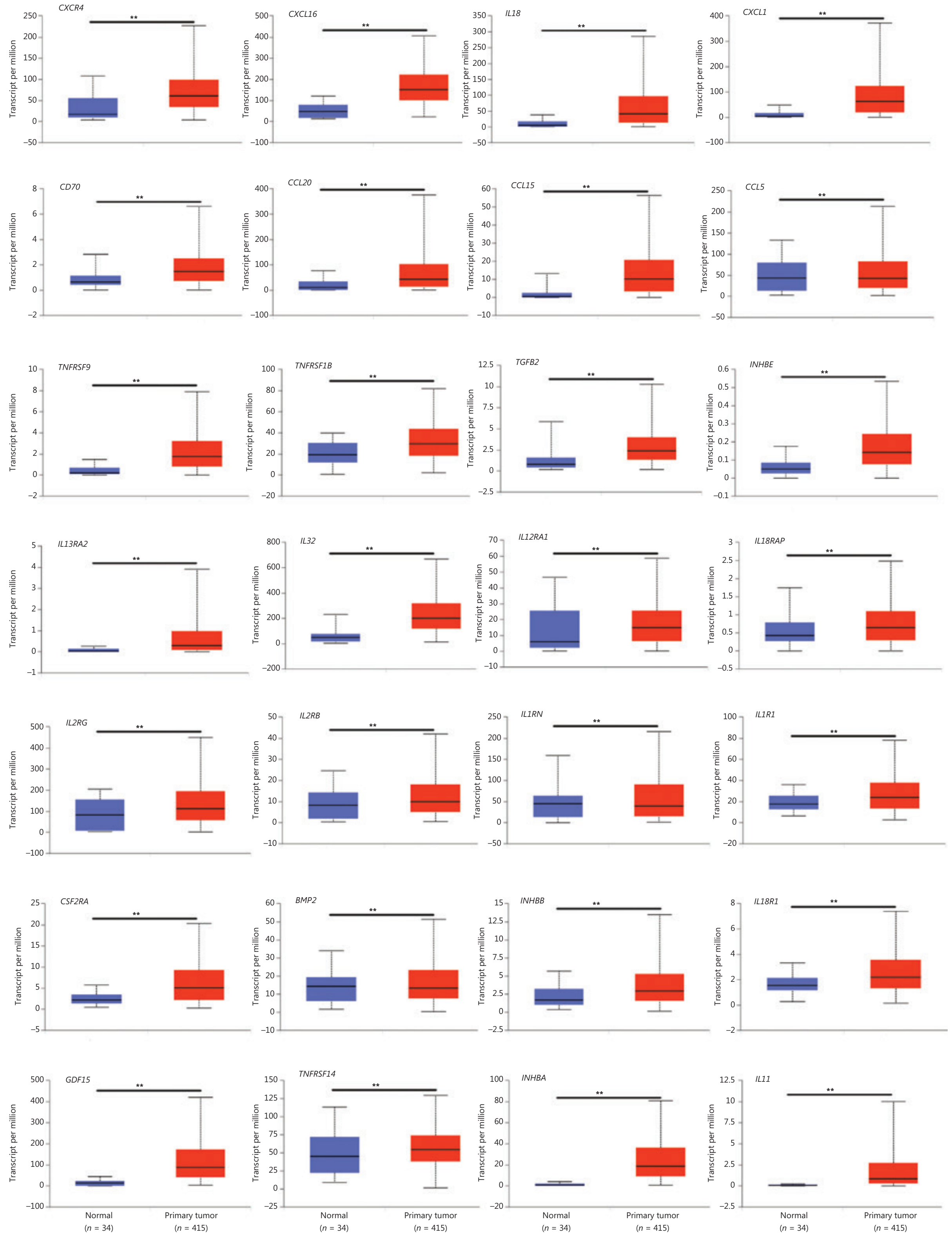

Figure S8 The expressions of *BMP2*, *CCL5*, *CCL15*, *CCL20*, *IL8*, *CD70*, *CSF2RA*, *CXCL1*, *CXCL16*, *CXCR4*, *TGFB2*, *GDF15*, *IL11*, *IL1R1*, *IL1RN*, *IL13RA2*, *IL32*, *IL2RB*, *IL2RG*, *IL22RA1*, *IL18R1*, *IL18RAP*, *INHBA*, *INHBB*, *INHBE*, *TNFRSF9*, *TNFRSF1B*, and *TNFRSF14* in stomach adenocarcinomas (STAD) in the Ualcan database (**$P < 0.01$).

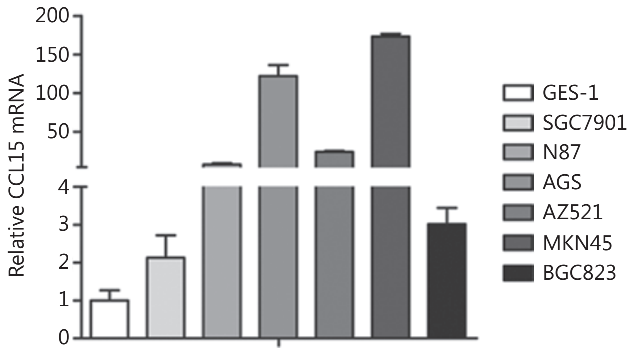

Figure S9 The relative mRNA expression levels of *CCL15* in a normal gastric epithelial cell line (GES-1) and gastric cancer cell lines (SGC7901, N87, AGS, AZ521, MKN45, and BGC823) was detected by qRT-PCR (n = 3). *GAPDH* was used as an internal control.

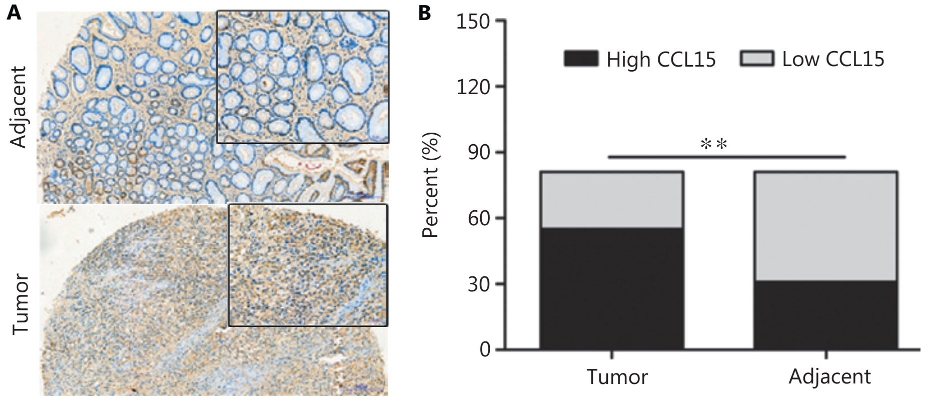

Figure S10 The expression of *CCL15* in a gastric cancer tissue microarray. (A) The expression of *CCL15* was examined in tumor and non-tumor adjacent tissues by immunohistochemical (IHC) analysis using a gastric cancer tissue microarray. Scale bars = 200 µm and 50 µm. (B) *CCL15* staining in tumor and adjacent non-tumor tissues was semiquantitatively calculated according to the staining intensity and percentage of positive cells (**P < 0.01). IHC scores < 6 and ≥ 6 were considered low and high expressions, respectively.

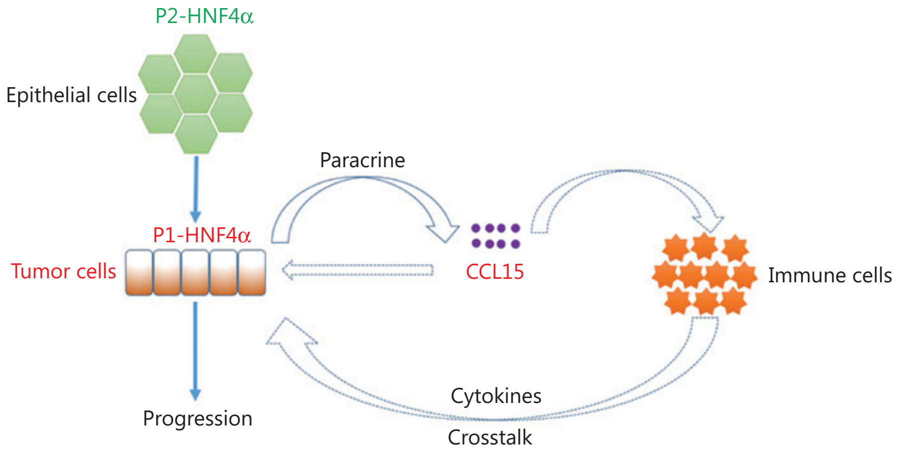

Figure S11 Schematic model of the proposed theory in this study.
